# Regulation of Axon Guidance by Slit2 and Netrin-1 Signaling in the Lacrimal Gland of *Aqp5* Knockout Mice

**DOI:** 10.1167/iovs.64.12.27

**Published:** 2023-09-14

**Authors:** Ying Bai, Guohu Di, Huanhuan Ge, Bin Li, Kaier Zhang, Di Zhang, Dianqiang Wang, Peng Chen

**Affiliations:** 1 Department of Human Anatomy, Histology and Embryology, School of Basic Medicine, Qingdao University, Qingdao, China; 2 Institute of Stem Cell Regeneration Medicine, School of Basic Medicine, Qingdao University, Qingdao, China; 3 Department of Ophthalmology, Qingdao Aier Eye Hospital, Qingdao, China

**Keywords:** lacrimal gland (LG), aquaporin 5 (AQP5), axon guidance, dry eye disease (DED)

## Abstract

**Purpose:**

Dry eye disease (DED) is multifactorial and associated with nerve abnormalities. We explored an Aquaporin 5 (AQP5)-deficiency-induced JunB activation mechanism, which causes abnormal lacrimal gland (LG) nerve distribution through Slit2 upregulation and Netrin-1 repression.

**Methods:**

Aqp5 knockout (*Aqp5^−^^/^^−^*) and wild-type (*Aqp5^+/+^*) mice were studied. LGs were permeabilized and stained with neuronal class III β-tubulin, tyrosine hydroxylase (TH), vasoactive intestinal peptide (VIP), and calcitonin gene-related peptide (CGRP). Whole-mount images were acquired through tissue clearing and 3D fluorescence imaging. Mouse primary trigeminal ganglion (TG) neurons were treated with LG extracts and Netrin-1/Slit2 neutralizing antibody. Transcription factor (TF) prediction and chromatin immunoprecipitation-polymerase chain reaction (ChIP-PCR) experiments verified the JunB binding and regulatory effect on Netrin-1 and Slit2.

**Results:**

Three-dimensional tissue and section immunofluorescence showed reduced LG nerves in *Aqp5^−^^/^^−^* mice, with sympathetic and sensory nerves significantly decreased. Netrin-1 was reduced and Slit2 increased in *Aqp5^−^^/^^−^* mice LGs. *Aqp5^+/+^* mice LG tissue extracts (TEs) promoted *Aqp5^−^^/^^−^* TG neurons axon growth, but Netrin-1 neutralizing antibody (NAb) could inhibit that promotion. *Aqp5^−^^/^^−^* mice LG TEs inhibited *Aqp5^+/+^* TG axon growth, but Slit2 NAb alleviated that inhibition. Furthermore, JunB, a Netrin-1 and Slit2 TF, could bind them and regulate their expression. SR11302, meanwhile, reversed the Netrin-1 and Slit2 shifts caused by AQP5 deficiency.

**Conclusions:**

AQP5 deficiency causes LG nerve abnormalities. Persistent JunB activation, the common denominator for Netrin-1 suppression and Slit2 induction, was found in *Aqp5^−^^/^^−^* mice LG epithelial cells. This affected sensory and sympathetic nerve fibers’ distribution in LGs. Our findings provide insights into preventing, reversing, and treating DED.

Dry eye disease (DED), a multifactor disorder, is characterized by persistent tear film instability or absence.^1^ Reduced tear secretion and evaporation of tears from the ocular surface are the main causes of tear film instability.^2^^,^^3^ The lacrimal gland functional unit (LFU), including the lacrimal gland (LG) and related nervous system, mainly regulates the production, transport, and clearance of tears to maintain the homeostasis of the ocular surface.^4^^,^^5^ Neuromodulation of the LG is a key factor affecting tear secretion.^6^

There are three kinds of LG nerve fibers. First, sensory nerve fibers, on the ophthalmic branch of the trigeminal nerve, form a neural network around ducts and acinar cells of the LG and supply the LG, lateral conjunctiva, and eyelid skin. Second, sympathetic nerve fibers, which are branches of the internal carotid plexus, regulate the normal secretion of the LG. Third, parasympathetic nerve fibers, which are branches of the facial nerve, function to control the secretion of the LG, too.^7^^,^^8^ When afferent sensory nerves in the cornea and conjunctiva are activated, efferent parasympathetic and sympathetic nerves innervating the LG are stimulated.^9^ Then, the release of neurotransmitters causes the LG to secrete tears that reach the ocular surface.^10^^,^^11^

Aquaporin 5 (AQP5), a member of the aquaporin family, is responsible for transporting water and has also been reported to promote hydrogen peroxide permeation.^12^ AQP5 is mainly distributed in the acinar apical membrane and duct cells in both mouse and human LGs.^13^^,^^14^ It has been shown that AQP5 expression is reduced in the LGs in mouse models of Sjogren's disease or DED.^15^^,^^16^ It has also been reported that AQP5 and muscarinic receptor type 3 (M3R) transmit the parasympathetic nervous system.^17^ Previous studies have found that the upregulation of AQP5 in the sciatic nerve of mice and rats after nerve injury suggests a possible role for AQP5 in promoting nerve regeneration following injury.^18^ AQP5 facilitates the nerve regeneration of the cornea by reactivating the Akt signaling pathway.^19^ Furthermore, the FoxO1–AQP5 axis alleviates chronic constriction injury (CCI)-induced neuropathic pain (NP) by modulating the ERK and p38 MAPK signaling pathways.^20^ However, the effect and mechanism of AQP5 on the LG nerve remain unclear.

Axon guidance refers to mechanisms that allow developing axons to extend from the neuron body and reach the target tissues. A nascent axonal growth cone can integrate and transduce many different stimuli received from the surrounding extracellular environment. That supports the precise and predictable shaping of the axonal routes in the developing nervous system.^21^ At the tip of an axon, there are many axon guidance cues that can be divided into repellent or attractant.^22^ According to several recent studies, the creation of new nerve bridge tissue and accurate axon regeneration following peripheral nerve transection injury are controlled by the signaling proteins Netrin-1, Slit3, and EphrinB2.^23^ Furthermore, inhibition of RGMa, a kind of axon guidance cue, alleviates symptoms of neuromyelitis optica (NMO) related to AQP4.^24^^,^^25^

In a previous study, we found that *Aqp5^−^^/^^−^* mice had a stable phenotype of DED from birth,^19^^,^^26^ and the regeneration of reactive oxygen species (ROS) induced by AQP5 deficiency promoted NLRP3 inflammasome activation in LG epithelial cells.^27^ Our results showed that the distribution of sensory and sympathetic nerves was reduced in the LGs of *Aqp5^−^^/^^−^* mice. Simultaneously, this was accompanied by downregulation of Netrin-1 expression and upregulation of Slit2 expression. Moreover, AQP5 deficiency caused upregulation of JunB in LGs. By using RNA sequencing and a chromatin immunoprecipitation-polymerase chain reaction (ChIP-PCR) assay, we uncovered the transcription factor (TF) JunB, a member of the AP-1 family, as the common denominator for Netrin-1 suppression and concomitant Slit2 induction in LG epithelial cells. These data imply that AQP5-deficiency-induced persistent JunB activation drives changes in the distribution of nerve fibers in the LG. Our findings offer theoretical support for the development of strategies to effectively prevent, reverse, and treat aqueous tear-deficient dry eye.

## Methods

### Animals

*Aqp5^−^^/^^−^* mice were identified through previous studies as appropriate research subjects and thus were obtained for this study, along with C57BL/6 mice, all 3 months old. The experimental protocols were approved by the Ethics Committee of the Medical College of Qingdao University.

Fluoro-Gold (FG, 2% in saline; Fluorochrome, LLC, Denver, CO, USA) was used as a retrograde tracer to identify neurons in the LG and TG to detect the origin of axon after subconjunctival injection with FG.

### Whole-Mount Immunostaining and Tissue Clearing

The mice were given 4% isoflurane in oxygen to induce anesthesia before being cervically dislocated and euthanized. The LG was dissected and fixed in 4% polyformaldehyde. The tissues were washed twice in PBS. Then, the tissues were permeabilized by washing them in 50% methanol in PBS, 80% methanol in deionized water, and, finally, 100% methanol. Next, we washed the tissues in 20% methanol, then 80% methanol in deionized water, followed by 50% methanol in PBS, then 100% PBS, and, finally, PBS with 2% Triton X-100. We blocked the tissues in goat serum (BOSTER, Boster Bio-Technology, Wuhan, China). The tissues were then incubated with the indicated neuronal class III β-tubulin (Tubulin βIII; #845502; BioLegend, San Diego, CA, USA), tyrosine hydroxylase (TH; #AB152; Millipore, Bedford, MA, USA), vasoactive intestinal peptide (VIP; #ab272726; Abcam, Cambridge, MA, USA) or calcitonin gene-related peptide (CGRP; #283568; Abcam), which were diluted in PBS/0.2% Tween-20/5% DMSO/3% goat serum. The incubation occurred at room temperature with gentle shaking, and following that, the tissues were washed in PBS. The tissues were then incubated with the indicated Alexa FluorTM 488 IgG (H+L; Invitrogen, Carlsbad, CA, USA) in PBS at room temperature overnight and washed in PBS before the tissue clearing began.

Immunolabeled LGs were treated with 50% ethanol in PBS, 80% ethanol in deionized water, and, finally, 100% ethanol with gentle shaking. We removed the tissues from the ethanol and ensured the ethanol was absorbed with a paper towel. We added CytoVistaTM Tissue Clearing Reagent (#V11304; Invitrogen, Thermo Fisher Scientific, Waltham, MA, USA) to completely cover the LGs and then we incubated them at 37°C. Last, we transferred the LGs to CytoVistaTM Tissue Clearing Enhancer (#V11302; Invitrogen) to finish the clearing process at 4°C.

### 3D Lightsheet Imaging

The immunolabeled and optically cleared LG tissues were imaged using a STELLARIS 5 confocal microscope (Leica, Germany). The tissues were immersed in the imaging chamber filled with CytoVistaTM Tissue Clearing Enhancer. For imaging at 10 × magnification, each tissue was scanned using a light sheet from the bottom side with a step size of 2 µm. Then, 2.5 × magnified images were captured using a single light sheet.

Imaris (http://www.bitplane.com/imaris/imaris) was used to 3D reconstruct the image stacks obtained from the light sheet imaging. Orthogonal projections were generated to create the representative 3D images shown in the figures. To quantify innervations in the LG, four 500 µm × 500 µm × 100 µm cubic volumes were randomly selected from the reconstructed 3D images of each tissue. Neuro-positive axons in each cubic volume were manually traced.

### RNA Sequencing and Data Analysis

Total RNA was isolated from LG tissue. The RNA quality was then assessed with the NanoDrop ND-1000 (Thermo Fisher Scientific, Waltham, MA, USA). Denaturing agarose gel electrophoresis was used to assess RNA integrity and gDNA contamination. Cloud-Seq Biotech (Shanghai, China) did the RNA high throughput sequencing. Following the manufacturer's instructions, total RNA was utilized to remove the rRNAs using the NEBNext rRNA Depletion Kit (New England Biolabs, Inc., Ipswich, MA, USA). RNA libraries were created by following the manufacturer's instructions with the NEBNext UltraTM II Directional RNA Library Prep Kit (New England Biolabs, Inc.). The BioAnalyzer 2100 system (Agilent Technologies, Inc., Santa Clara, CA, USA) was used to control and quantify the libraries. With 150 bp paired end reads, library sequencing was performed on an Illumina Hiseq device.

Hisat2 software (version 2.0.4) was used to match the high-quality reads to the mouse reference genome (UCSC MM10). The cuffdiff software (version 2.2.1, part of cufflinks) was then used to obtain the FPKM as the expression profiles of mRNA, and fold change and *P* value were calculated based on FPKM, and differentially expressed mRNA were detected.

### Culture and Treatment of Primary LG Epithelial Cells

The LGs were dissected from 3-month-old mice and placed in PBS containing 1% penicillin–streptomycin (Servicebio, Wuhan, China). After washing with PBS twice, LGs were cut and digested in MEM-ALPHA medium containing type II collagenase (Gibco, Waltham, MA, USA) at 37°C with 50 revolutions per minute (rpm) shaking for 30 minutes. The cell suspension was centrifuged at 1000 rpm, and the cell pellet was resuspended in DMEM (Gibco). After centrifugation at 1000 rpm for 5 minutes, the LG epithelial cells were resuspended in DMEM/high-glucose medium (cytiva, Marlborough, MA, USA) containing 20% fetal bovine serum (FBS) and 1% penicillin–streptomycin. Next, 48-well plates were used to culture the cells. The whole medium was changed every other day until 80% of the area was taken up by cells.

Tissue extracts (TEs) were obtained by taking the supernatant from the lysate of LG tissues. TEs of LGs, neutralizing antibodies (NAbs), and recombinant factors (all from R&D System, Minneapolis, MN, USA), were mixed with medium to culture cells, and the same volume of PBS was used as a control. SR11302 (#T23384, TargetMol; TOPSCIENCE, Shanghai, China) was added to the cells at 10 µM, and an equal volume of DMSO was added to the control group. Proteins were collected after 24 hours.

### Culture of Primary Trigeminal Ganglion Neurons 

The trigeminal ganglions (TGs) were dissected from the mice and placed in ice-cold Hanks’ balanced salt solution (HBSS). After cutting up the TGs, we added them to HBSS containing 67 mg/mL cysteine, 0.2% saturated sodium bicarbonate solution, and 2 mg/mL papain at 37°C. Then, HBSS containing type II collagenase was used to digest trigeminal ganglion neuron (TGN) cells. Percoll density gradient centrifugation was performed on the cell suspension. The cells were planted in Leibovitz's L-15 medium (Gibco) containing 10% FBS and 1% penicillin–streptomycin in a 48-well plate already coated with laminin (BioLamina, Sundbyberg, Sweden). This was transferred to a cell incubator, and we added Neurobasal-A medium (Gibco) with 2% B27 (Gibco) after 3 hours.

### Quantitative Real-Time PCR 

FastPure Cell/Tissue Total RNA Isolation Kit version 2 (Vazyme, Nanjing, China) was used to extract the total RNA. The cDNA was synthesized with a PrimeScript RT Reagent kit (TaKaRa, Kusatsu, Japan). Quantitative real-time PCR (qRT-PCR) was performed using ChamQ SYBR Color qPCR Master Mix (Vazyme). The reaction products were analyzed using Bio-Rad CFX96 Real-Time Systems (Bio-RAD, Hercules, CA, USA). All primers used in the qRT-PCR are shown in the Table.

**Table.** Gene-Specific Primers Used in This Study

Gene Name	Primer Pair		Application
Netrin-1	Forward	CCTCCAAAGGCAAGCTGAAG	qPCR
	Reverse	TACGACTTGTGCCCTGCTTGT	
Slit2	Forward	GCGAGTTCGAGCCAGCTATG	
	Reverse	ACTTTAGGGCTTCCTCCATCCA	
Netrin-1	Forward	AAGGAGAACAAAGGACTCATTCAAG	ChIP-PCR
	Reverse	AGGAGGCAGAAGCAGGTAGATCT	
Slit2	Forward	CGCGACTCAAAAAGCTACAACA	
	Reverse	GGTCTCCTCCACACAACTCCTT	
JunB	Forward	CTGTGTCCCCCATCAACATG	qPCR
	Reverse	GCGTTCTCAGCCTTGAGTGTCT	
c-Jun	Forward	CCCCTATCGACATGGAGTCTCA	
	Reverse	CGGAGTTTTGCGCTTTCAAG	
JunD	Forward	GTCGCCCATCGACATGGA	
	Reverse	CTCGGTGTTCTGGCTTTTGAG	
Fra-1	Forward	GACCGACAAATTGGAGGATGAG	
	Reverse	CCAGAACCACCTGGGTCCTT	

### Immunofluorescence Staining

The cells or frozen sections were fixed in 4% paraformaldehyde at room temperature. Then, 5% bovine serum albumin (BSA) was used to incubate them. The samples were incubated overnight at 4°C with the Netrin-1 (#YT3042; Immunoway, Plano, TX, USA) or Slit2 (#A3467; ABclonal, Wuhan, China) antibodies and then for 1 hour at room temperature with 488-labeled secondary antibody in the dark. The 4′, 6-diamidino-2-phenylindole (DAPI; Beyotime, Shanghai, China) solution was used to stain the cell nucleus. Staining photographs were taken using an upright fluorescence microscope (Nikon Ni-U; Nikon, Tokyo, Japan) and an inverted microscope (Nikon Ti2-U; Nikon).

### Western Blot Analysis and Antibodies

The cells and tissues were collected, lysed in lysis buffer, and broken by sonication. Then, the samples were centrifuged at 12,000 rpm to collect the supernatant. The proteins were separated by 7.5% to 12.5% SDS–polyacrylamide gel and electro-transferred onto polyvinylidene fluoride (PVDF) membranes (Millipore). The membranes were blocked with 3% non-fat milk in Tris-buffered saline-Tween 20 (TBST) at room temperature and incubated with the primary antibody overnight at 4°C. Then, the samples were incubated with the respective second antibody for 1 hour. The protein signal was visualized using an enhanced chemiluminescence kit (Meilunbio, Dalian, China). β-actin was used as the internal control. For each sample, the levels of protein of interest were normalized to the level of β-actin. Primary antibodies included anti-Tubulin βIII antibody, anti-TH antibody, anti-VIP antibody, anti-CGRP antibody, anti-β-actin antibody (#AC026; ABclonal), anti-Netrin-1 antibody (#A16236; ABclonal), anti-Slit2 antibody (#ab246503; Abcam), and anti-JunB antibody (#A5290, ABclonal).

### Chromatin Immunoprecipitation Assay

A ChIP assay was performed according to the instructions of the BeyoChIP Enzymatic ChIP Assay Kit (#P2083S; Beyotime, Shanghai, China). Briefly, isolated primary LG epithelial cells were finely homogenized in 1% formaldehyde and then crosslinked at 37°C. The process of cross-linking was terminated by adding glycine solution at room temperature. Then, the cells were washed and collected with PMSF (1 µM). After the lysed cells were enzymatic, the fragments of chromatin (some for input) were incubated with Protein A/G Magnetic Beads/Salmon Sperm DNA and separated on a magnetic frame. Next, 2 µg of antibody against JunB (C-11) X (SC-8051; Santa Cruz Biotechnology, Santa Cruz, CA, USA) was added to the supernatant and incubated at 4°C overnight. The Protein A/G Magnetic Beads/Salmon Sperm DNA was added to the mixture. Then, the pellets were treated with elution buffer, and the supernatant was obtained after magnetic separation. For 2 hours, 5M NaCl was used for decrosslinking at 65°C. DNA was purified by phenol and chloroform extraction. The purifying DNA fragments were amplificated by nested PCR, and then the PCR products were subjected to electrophoresis in 1.5% agarose gel. The primers for ChIP are shown in the Table.

### Quantification and Statistical Analysis

GraphPad Prism (http://www.graphpad.com/scientific-software/prism) was used to analyze the data. All results are shown as the mean ± standard deviation. The distribution of the data was checked for normality using the Shapiro-Wilk test. If groups passed the normality test, Student's unpaired *t*-test was used. Otherwise, the Mann-Whitney test was used to analyze the difference between two groups. The *P* values of less than 0.05 were deemed significant (*P < 0.05, **P < 0.01, and ***P < 0.001). The statistical details of the experiments can be found in the figure legends.

## Results

### Neural Changes in the *Aqp5^−^^/^^−^* Mouse LGs

**Figure 1.**
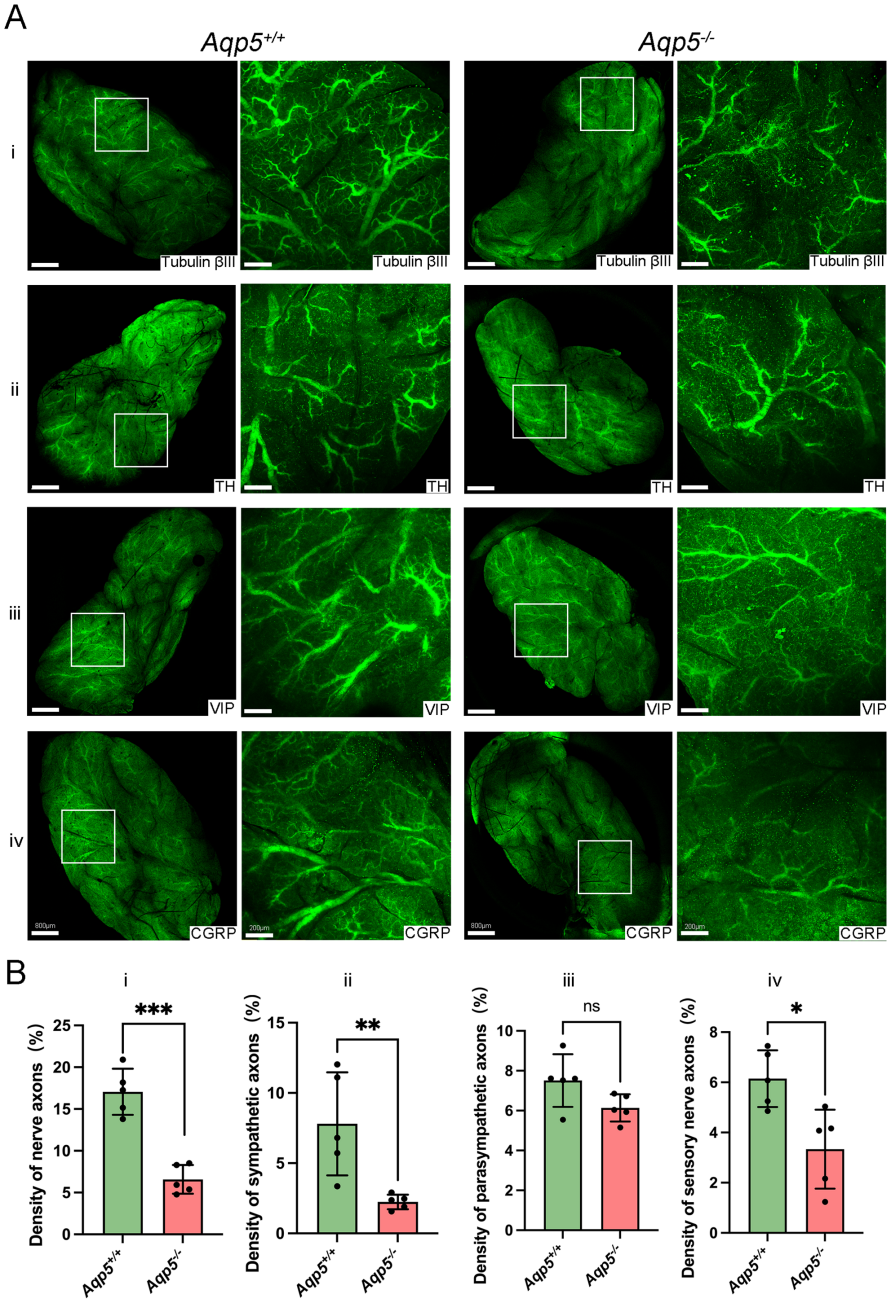
**Abnormal LG nerves in *Aqp5*^−^^/−^ mice.** (**A**) The unsectioned LGs of adult mice and *Aqp5*^−/−^ mice were processed for tubulin βIII, TH, VIP, and CGRP immunolabeling. The 3D projection image of tissue at 2.5 times (*left panel*) and 10 times (*right panel*) magnification of the light sheet image are shown. Scale bars = 800 µm (2.5x) and 200 µm (10x). (**B**) The ratio of nerve volume to total volume of *Aqp5*^+/+^ and *Aqp5*^−/−^ lacrimal gland was quantified analysis (*n* = 5; mean ± SD; n.s., not significant; *P < 0.05; **P < 0.01; Student's *t*-test).

We identified AQP5 expression in the LG of *Aqp5^+/+^* and *Aqp5^−^^/^^−^* mice by immunofluorescence (Supplementary Fig. S1). The LGs were studied using immunolabeling-enabled three-dimensional imaging of solvent-cleared organs plus (iDISCO+). We first examined the mouse LGs by immunolabeling Tubulin βIII, a special pan-neural marker, which showed the 3D neural distribution (Fig. 1Ai, Supplementary Videos S1, S2). In *Aqp5^−^^/^^−^* LGs, the nerve branches became thinner and sparser and assumed a more disordered arrangement than for *Aqp5^+/+^* LGs. The neural distribution of LGs was reduced in *Aqp5^−^^/^^−^* mice compared to *Aqp5^+/+^* mice (*Aqp5^+/+^* 17.06 ± 1.232%, *Aqp5^−^^/^^−^* 6.581 ± 0.7688%, *P* < 0.01; Fig. 1Bi). Sympathetic neuron marker TH, parasympathetic neurons marker VIP, and sensory neuron marker CGRP were respectively immunolabeled in mice LGs (Fig. 1Aii–iv, Supplementary Videos S3–S8). The fluorescence-positive percentages of TH and CGRP were significantly decreased in *Aqp5^−^^/^^−^* mice compared to *Aqp5^+/+^* mice (TH: *Aqp5^+/+^* 7.802 ± 1.641%, *Aqp5^−^^/^^−^* 2.241 ± 0.2319%, *P* < 0.01; CGRP: *Aqp5^+/+^* 6.149 ± 0.505%, *Aqp5^−^^/^^−^* 3.336 ± 0.7038%, *P* < 0.05; Fig. 1Bii, iv). In contrast, the immunolabeling of VIP revealed no difference between *Aqp5^+/+^* and *Aqp5^−^^/^^−^* (*Aqp5^+/+^* 7.509 ± 0.5898% and *Aqp5^−^^/^^−^* 6.139 ± 0.3062%, *P* = 0.0732; Fig. 1Biii). The findings suggest a general decrease in nerve density within the LGs of *Aqp5^−^^/^^−^* mice when compared to those of *Aqp5^+/+^* mice.

### Decreased Sympathetic and Sensory Nerve Density in *Aqp5^−^^/^^−^* Mouse LGs

**Figure 2.**
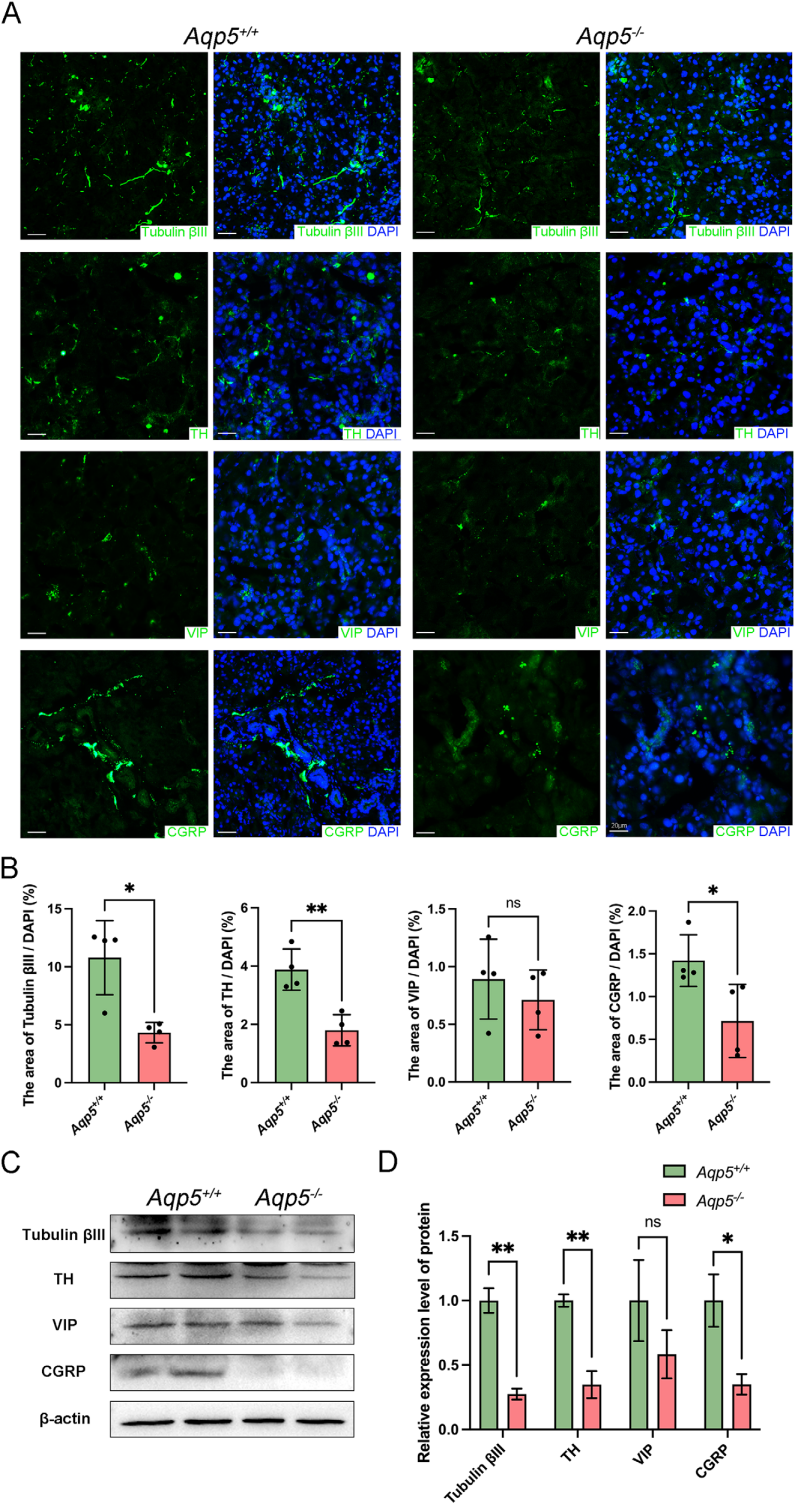
**Lower sympathetic and sensory nerve densities in the LG of *Aqp5^−^^/^^−^* mice.** (**A**) Representative photomicrographs of the LGs from adult *Aqp5*^+/+^ and *Aqp5*^−/−^ mice immunostained for tubulin βIII, TH, VIP, CGRP (*green*) and DAPI-stained cell nuclei (*blue*). Scale bars = 20 µm. (**B**) Positive analyses of immunofluorescence staining presented in (**A**). The significance of the difference between *Aqp5*^+/+^ and *Aqp5*^−/−^ mice in respective nerve group is presented (*n* = 4; mean ± SD; n.s., not significant; *P < 0.05; **P < 0.01; Mann-Whitney test). (**C**) Western blot analysis of content of protein of tubulin βIII, TH, VIP, and CGRP in LG tissues lysates from *Aqp5*^+/+^ and *Aqp5*^−/−^ adult mice. (**D**) Densitometric analyses of Western blots presented in (**C**). The significance of the difference between *Aqp5*^+/+^ and *Aqp5*^−/−^ mice in respective nerve group is presented. Data are represented as means ± SD (n.s., not significant; *P < 0.05; **P < 0.01; Student's *t*-test).

Sections of LGs were immunolabeled with tubulin βIII, TH, VIP, and CGRP (Fig. 2A). Analyses of fluorescence-positive LG sections showed that tubulin βIII significantly decreased in *Aqp5^−^^/^^−^* LGs (*Aqp5^+/+^* 10.79 ± 1.596% and *Aqp5^−^^/^^−^* 4.321 ± 0.4410%, *P* < 0.01; Fig. 2B). Less Fluoro-Gold-labeled cell bodies were observed in LGs and TGs of *Aqp5*^−/−^ mice, compared to that of *Aqp5*^+/+^ mice (Supplementary Fig. S2) Compared to *Aqp5^+/+^* LGs, *Aqp5^−^^/^^−^* LGs expressed a lower density of TH (*Aqp5^+/+^* 3.879 ± 0.3535% and *Aqp5^−^^/^^−^* 1.796 ± 0.2674%, *P* < 0.01; see Fig. 2B) and CGRP (*Aqp5^+/+^* 1.421 ± 0.1504% and *Aqp5^−^^/^^−^* 0.7146 ± 0.2138%, *P* < 0.05; see Fig. 2B), whereas we found no significant difference in VIP in LGs between *Aqp5^−^^/^^−^* and *Aqp5^+/+^* mice (see Fig. 2B). Western blot analysis revealed that a deficiency in *Aqp5* decreased the expression of tubulin βIII, TH, and CGRP in LGs but had no effect on the VIP level (Figs. 2C, 2D). The distribution of sympathetic and sensory nerves is reduced in the LGs of *Aqp5^−^^/^^−^* mice compared to those of *Aqp5^+/+^* mice, whereas there is no change in parasympathetic nerve distribution.

### Netrin-1 Reduction and Slit2 Elevation in the LGs and Primary LG Epithelial Cells of *Aqp5^−^^/^^−^* Mice

**Figure 3.**
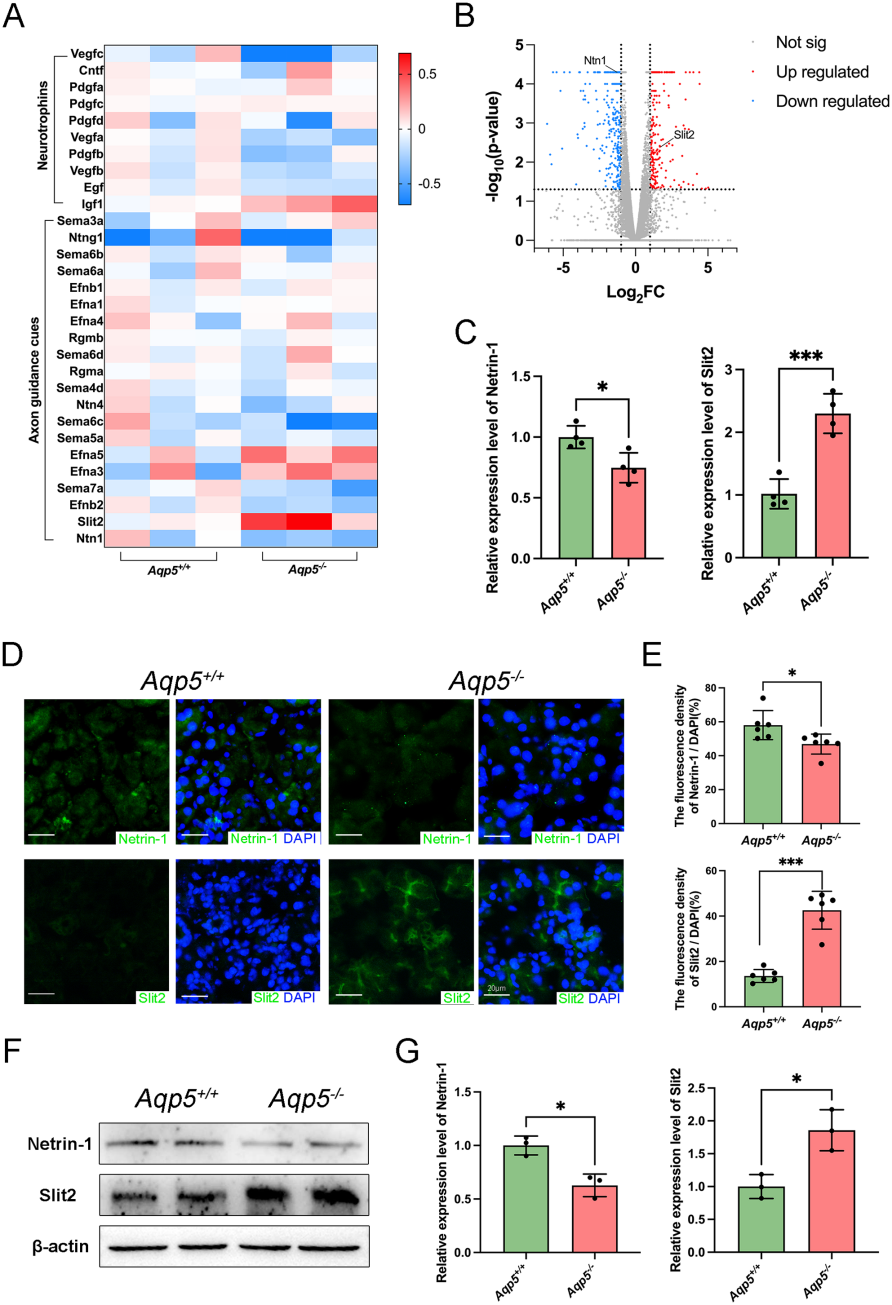
**Decreased Netrin-1 and increased Slit2 in the LG of *Aqp5*^−^^/^^−^** **mice.** (**A**) Heatmap analysis of mRNA profiles about main neurotrophins and axon guidance cues in LG of *Aqp5*^+/+^ and *Aqp5*^−/−^. (**B**) The volcano plot for all expressed genes. Differentially expressed genes (*P* < 0.05) between *Aqp5*^+/+^ and *Aqp5*^−/−^ LGs were presented with *blue* (*down*) and *red* (*up*). (**C**) Quantitative reverse transcription-polymerase chain reaction (qRT-PCR) analysis of the expression of Netrin-1 and Slit2 in LG tissues (*n* = 4; mean ± SD; *P < 0.05; *** *P* < 0.001). (**D**) Representative immunofluorescence images of LG tissue labeled with DAPI (*blue*), Netrin-1, and Slit2 (*green*). Scale bars = 20 µm. (**E**) Positive analyses of immunofluorescence staining presented in (**D**) (*n* = 6; mean ± SD; n.s., not significant; *P < 0.05; ***P < 0.001; Mann-Whitney test). (**F**) Western blot analysis of content of protein of Netrin-1 and Slit2 in LG tissues lysates from *Aqp5*^+/+^ and *Aqp5*^−/−^ adult mice. (**G**) Densitometric analyses of Western blots presented in (**F**) (*n* = 3; mean ± SD; *P < 0.05; Student's *t*-test).

To determine the reasons for the decreased distribution of sensory and sympathetic nerve fibers in the LGs of *Aqp5^−^^/^^−^* mice, we analyzed the expression of the main neurotrophic factor and axon guidance cues via RNA sequencing. Compared with *Aqp5^+/+^* mice, the expression of Slit2 (FC: 3.224, *P* = 0.004) was significantly increased, and the expression of Netrin-1 (Ntn1; FC: -2.167, *P* < 0.001) was significantly decreased in the LGs of *Aqp5^−^^/^^−^* mice (Fig. 3A). A volcano plot (Fig. 3B, Supplementary Fig. 3) revealed the DEGs in LGs between *Aqp5^+/+^* and *Aqp5^−^^/^^−^* mice. A total of 217 genes were markedly upregulated (including Slit2) and 282 markedly downregulated (including Ntn1) in LGs of *Aqp5^−^^/^^−^* mice compared to *Aqp5^+/+^* mice (Supplementary Table). The qRT-PCR results showed that the Netrin-1 expression level (*Aqp5^+/+^* 1.000 ± 0.0463 and *Aqp5^−^^/^^−^* 0.7475 ± 0.0617, *P* < 0.05) was lower and that of Slit2 (*Aqp5^+/+^* 1.018 ± 0.1183 and *Aqp5^−^^/^^−^* 2.298 ± 0.1575, *P* < 0.001) was higher in the LGs of *Aqp5^−^^/^^−^* compared to those of *Aqp5^+/+^* (Fig. 3C). Immunofluorescence staining revealed a lower Netrin-1 expression level (*Aqp5^+/+^* 58.05 ± 3.499% and *Aqp5^−^^/^^−^* 46.88 ± 2.415%, *P* < 0.05) and a higher Slit2 expression level (*Aqp5^+/+^* 13.56 ± 1.164% and *Aqp5^−^^/^^−^* 42.56 ± 3.406%, *P* < 0.001) in *Aqp5^−^^/^^−^* mice LG compared to *Aqp5^+/+^* mice (Figs. 3D, 3E). Western blot analysis showed that Netrin-1 expression decreased and Slit2 expression increased in the LGs of *Aqp5^−^^/^^−^* mice compared to *Aqp5^+/+^* (Figs. 3F, 3G).

**Figure 4.**
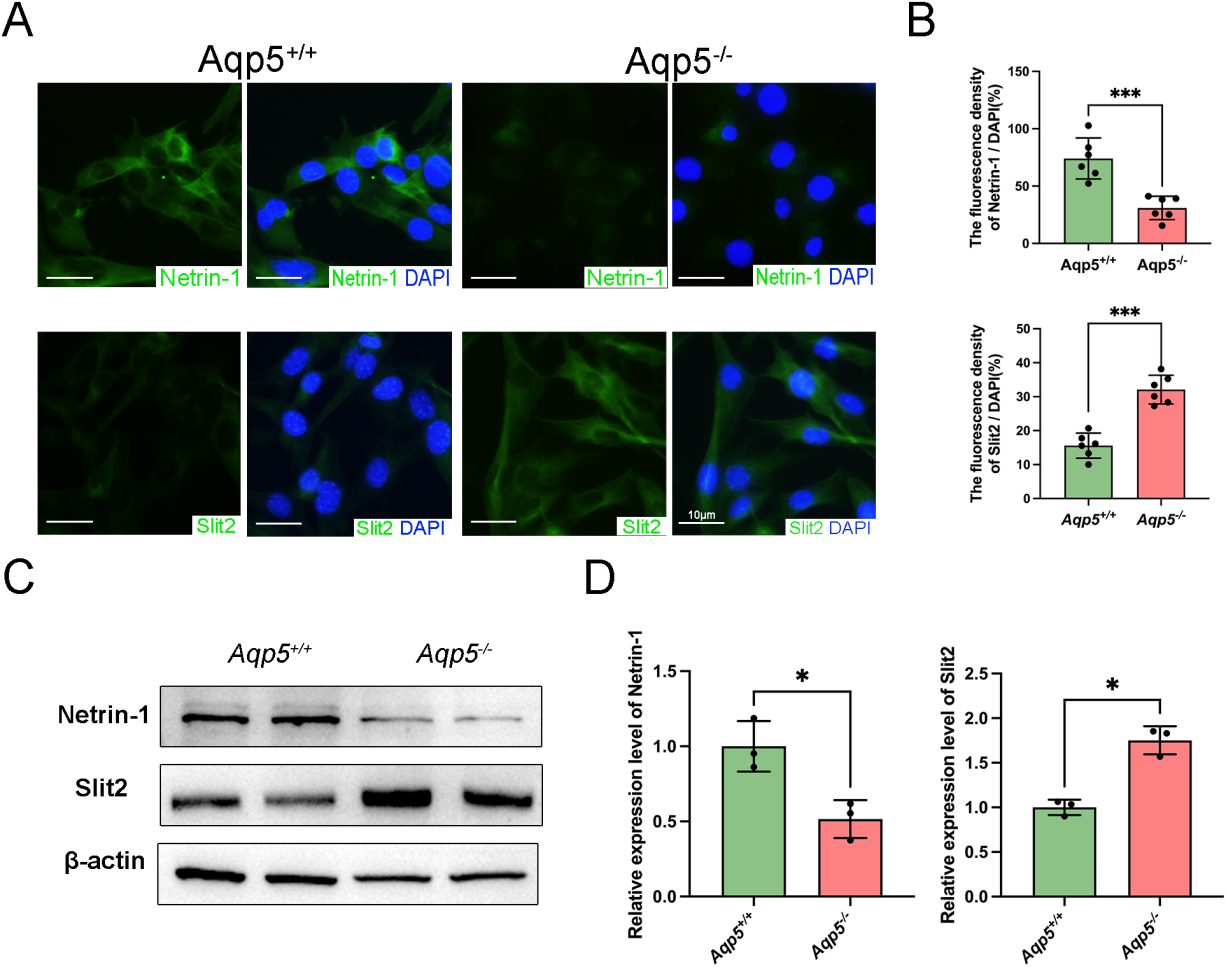
**Netrin-1 reduction and Slit2 elevation in primary LG epithelial cells of *Aqp5*^−^^/^^−^ mice.** (**A**) *Aqp5*^+/+^ and *Aqp5*^−/−^ LG epithelial cells were fixed and immunolabeled with Netrin-1 (*green*), Slit2 (*green*), and DAPI (*blue*). Scale bars = 10 µm. (**B**) Positive analyses of immunofluorescence staining presented in (**A**) (*n* = 5; mean ± SD; ***P < 0.001; Student's *t*-test). (**C**) Western blot analysis of content of protein of Netrin-1 and Slit2 in LG epithelial cells from *Aqp5*^+/+^ and *Aqp5*^−/−^ adult mice. (**D**) Densitometric analyses of Western blots presented in (**C**) (*n* = 3; mean ± SD; **P < 0.01; ***P < 0.001; Student's *t*-test).

The LG epithelial cells of *Aqp5^+/+^* and *Aqp5^−^^/^^−^* mice were isolated. Immunofluorescence staining showed that Netrin-1 was decreased (*Aqp5^+/+^* 74.10 ± 7.333 and *Aqp5^−^^/^^−^* 30.99 ± 4.166, *P* < 0.001) and Slit2 was increased (*Aqp5^+/+^* 15.58 ± 1.498 and *Aqp5^−^^/^^−^* 32.10 ± 1.729, *P* < 0.001) in the LG epithelial cells of *Aqp5^−^^/^^−^* mice compared to *Aqp5^+/+^* mice (Figs. 4A, 4B). Western blot analysis revealed that in LG epithelial cells, *Aqp5^−^^/^^−^* mice expressed less Netrin-1 and more Slit2 compared to *Aqp5^+/+^* mice (Figs. 4C, 4D). In the absence of AQP5, there is an upregulation of Slit2 and a downregulation of Netrin-1 in LG epithelial cells.

### TG Axonal Extension Regulated by Netrin-1 and Slit2

**Figure 5.**
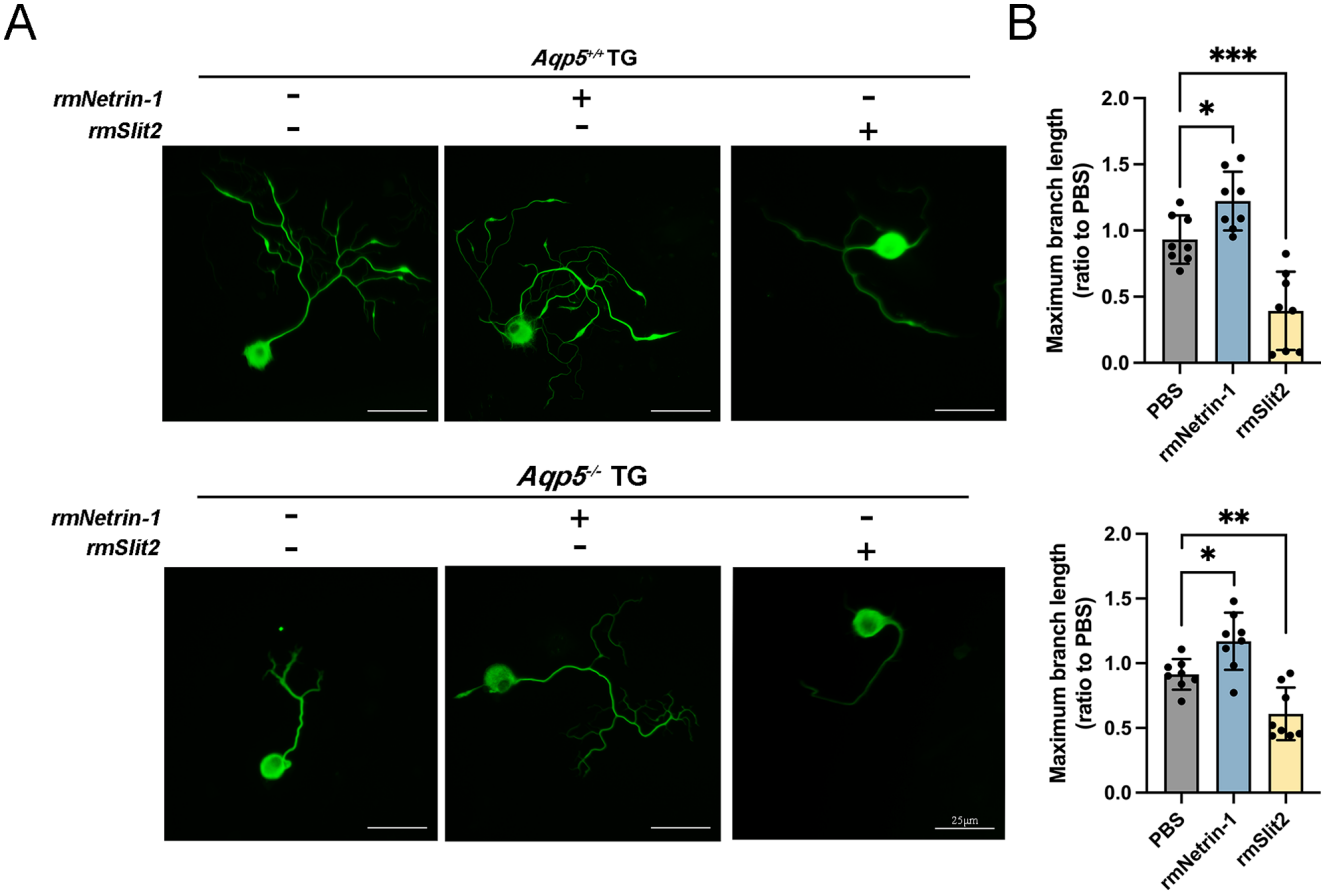
**Netrin-1 and Slit2 induced TG axonal extension and repulsion.** (**A**) Axonal growth of *Aqp5^+/+^* and *Aqp5^−^^/^^−^* TG neurons after addition of rmNetrin-1 and rmSlit2, respectively. Scale bars = 25 µm. (**B**) Quantitative statistics of maximum branch length of (**A**) (*n* = 8; mean ± SD; *P < 0.05; **P < 0.01; Mann-Whitney test).

To determine the effect of Netrin-1 and Slit2 on TG neurite growth, we treated TG neurons with recombinant factors. The maximum branch lengths of *Aqp5^+/+^* and *Aqp5^−^^/^^−^* LGs treated with rmNetrin-1 were significantly increased compared to those in the control group (Fig. 5A). In contrast with the control group, the maximum branch length of TGs with rmNetrin-1 increased to nearly 1.2 times the control length (*Aqp5^+/+^* 1.222 ± 0.2214 and *Aqp5^−^^/^^−^* 1.170 ± 0.2210; Fig. 5B). Meanwhile, the maximum branch lengths of *Aqp5^+/+^* and *Aqp5^−^^/^^−^* LGs treated with rmSlit2 were significantly reduced by approximately 50% (*Aqp5^+/+^* 0.393 ± 0.2954 and *Aqp5^−^^/^^−^* 0.6088 ± 0.203; see Fig. 5B) compared to the control group. The axonal growth of TG neurons is facilitated by Netrin-1, but this process is hindered by Slit2.

### AQP5 Affects Axon Outgrowth of TG Neuron Through Netrin-1 and Slit2

**Figure 6.**
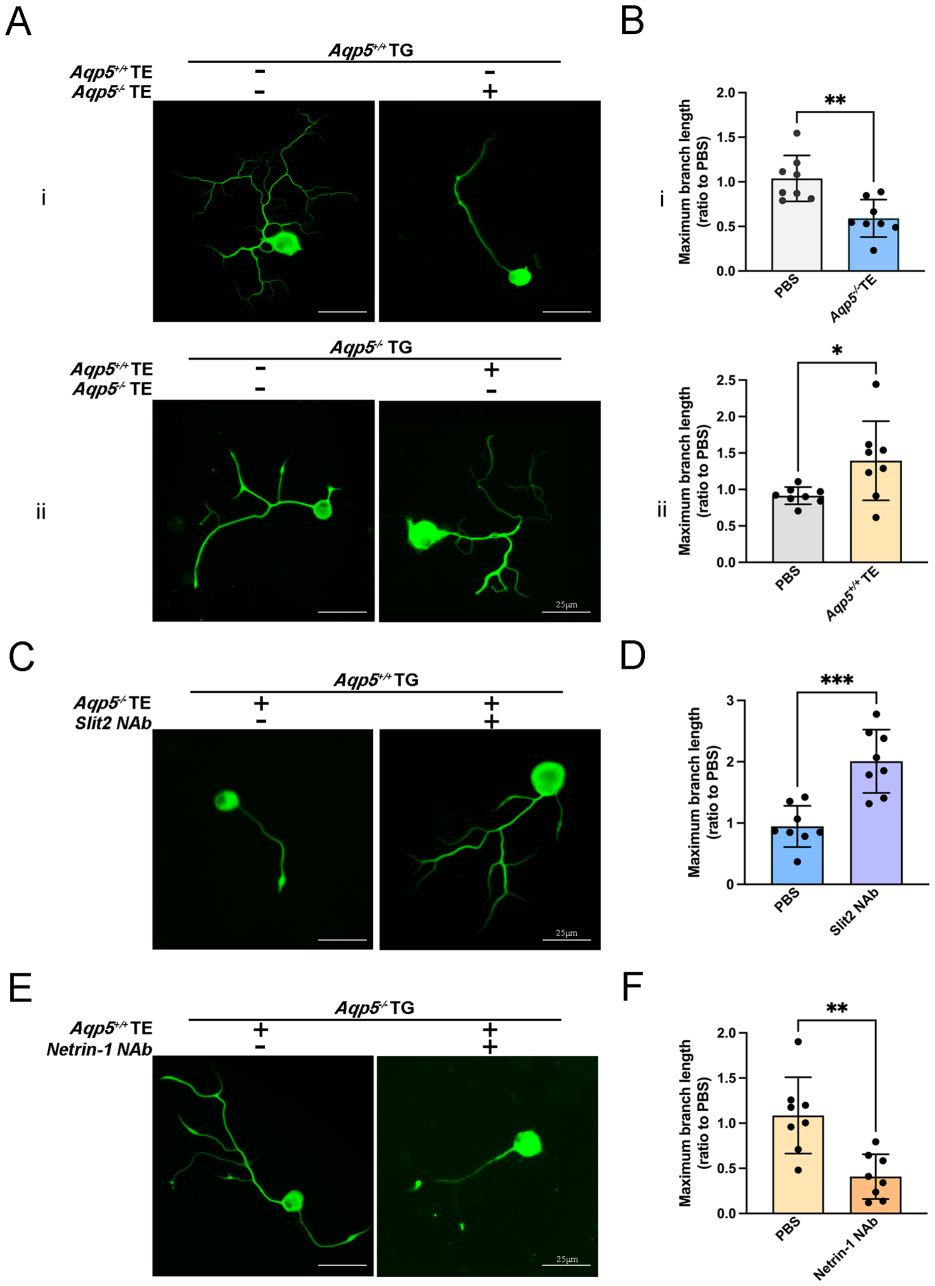
**The effect of TEs of LGs on the outgrowth of TG axons.** (**A**) Axonal growth of *Aqp5^+/+^* TG neurons after addition of tissue extractions from *Aqp5^−^^/^^−^* LGs and the Axon growth of *Aqp5^−^^/^^−^* TG cells after adding tissue extractions from *Aqp5^+/+^* LGs. (**B**) Quantitative statistics of maximum branch length of (**A**) (*n* = 8; mean ± SD; *P < 0.05; **P < 0.01; Student's *t*-test). (**C**) Axonal growth of *Aqp5^+/+^* TG cells after addition of TEs from *Aqp5^−^^/^^−^* LGs and Slit2 NAbs at the same time. (**D**) Quantitative statistics of maximum branch length of (**C**) (*n* = 8; mean ± SD; ***P < 0.001; Student's *t*-test). (**E**) Axonal growth of *Aqp5^−^^/^^−^* TG cells after addition of TEs from *Aqp5^+/+^* LGs and Netrin-1 NAbs at the same time. (**F**) Quantitative statistics of maximum branch length of (**E**) (*n* = 8; mean ± SD; **P < 0.01; Student's *t*-test). Scale bars = 25 µm.

The primary cultured TG sensory neurons of the *Aqp5^+/+^* and *Aqp5^−^^/^^−^* mice were treated with the TEs from LGs of *Aqp5^−^^/^^−^* and *Aqp5^+/+^*, respectively. After 24 hours, the TEs of *Aqp5^−^^/^^−^* LGs showed significantly slowed neurite growth of TG neurons when compared to the TEs of *Aqp5^+/+^* LGs (Fig. 6A, i). Statistical analysis showed that the maximum branch length of *Aqp5^+/+^* TG neurons treated with *Aqp5^−^^/^^−^* TEs was reduced by approximately half compared to control TG neurons (Fig. 6B, i). Meanwhile, the TEs of *Aqp5^+/+^* LGs demonstrated significantly accelerated neurite growth when compared to those of *Aqp5^−^^/^^−^* LGs (see Fig. 6A, ii). According to statistical analysis, the maximum branch length of *Aqp5^−^^/^^−^* TGs treated with *Aqp5^+/+^* TEs grew to 1.4 times that of the control group (see Fig. 6B, ii). The axonal growth of TG neurons was suppressed by the TEs derived from *Aqp5^−^^/^^−^* LGs, whereas it was enhanced by the TEs derived from *Aqp5^+/+^* LGs.

To screen out the factors that specifically stimulate or inhibit the axonal growth of TGs in the LGs of *Aqp5^+/+^* and *Aqp5^−^^/^^−^* mice, we added NAbs to the cell culture medium. The Slit2 NAb blocked the inhibitory effect of *Aqp5^−^^/^^−^* TEs on the axon growth of *Aqp5^+/+^* TG neurons, and it nearly doubled the maximum branch length (Figs. 6C, 6D). Meanwhile, the Netrin-1 NAb blocked the promoting effect of *Aqp5^+/+^* TEs on the axon growth of *Aqp5^−^^/^^−^* TG neurons, and it reduced the branch length of axons by more than half (Figs. 6E, 6F). The inhibitory effect of the TEs of *Aqp5*^−/−^ LGs on axonal growth of TG neurons was blocked by Slit2 NAb. Similarly, the promoting effect of the TEs of *Aqp5*^+/+^ LGs on axonal growth of TG neurons was blocked by Netrin-1 NAb.

### JunB Regulates the Expression of Netrin-1 and Slit2 in *Aqp5^−^^/^^−^* Mouse LGs

**Figure 7.**
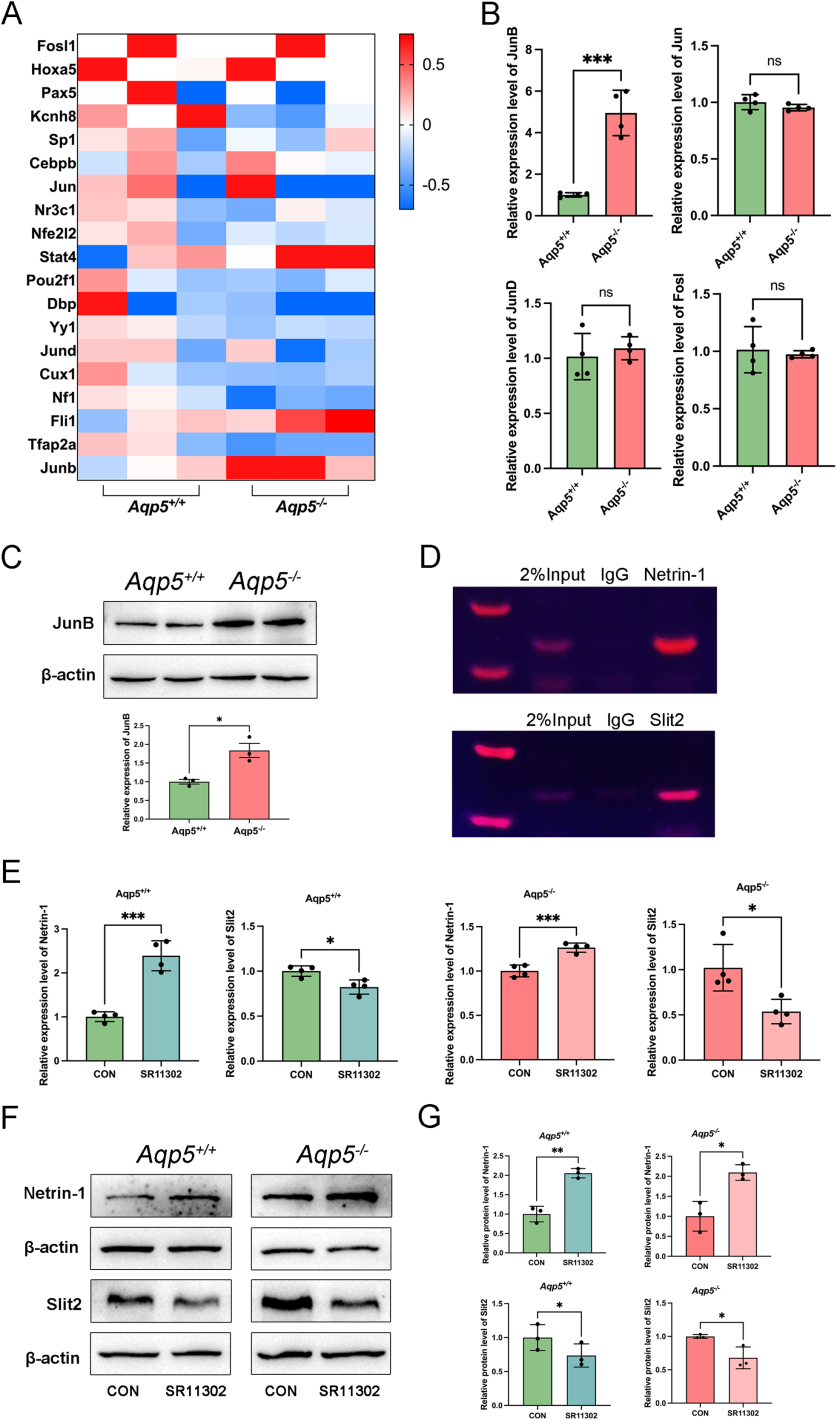
**JunB binds to and regulates Netrin-1 and Slit2 in *Aqp5^−^^/^^−^* mice LG epithelial cells.** (**A**) Heatmap analysis of mRNA profiles about transcription factors that co-transcribe Netrin-1 and Slit2 in LG of *Aqp5*^+/+^ and *Aqp5*^−/−^. (**B**) The qRT-PCR analysis of the expression of JunB, Jun, JunD, and Fosl in LG tissues (*n* = 4; mean ± SD; n.s., not significant; *** *P* < 0.001; Student's *t*-test). (**C**) Western blotting analysis showed the expression level JunB in LG epithelial cells. (**D**) ChIP-PCR analyses of JunB enrichment at binding sites in the murine Netrin-1 and Slit2 promoter. (**E**) The qRT-PCR analysis of the expression of Netrin-1 and Slit2 in *Aqp5^+/+^* and *Aqp5^−^^/^^−^* LG epithelial cells treated with SR11302 or DMSO 24 hours (*n* = 4; mean ± SD; * *P* < 0.05; *** *P* < 0.001; Student's *t*-test and Mann-Whitney test). (F) Western blot analysis of content of protein of Netrin-1 and Slit2 in *Aqp5^+/+^* and *Aqp5^−^^/^^−^* LG epithelial cells treated with SR11302 or DMSO 24 hours. (**G**) Densitometric analyses of Western blots presented in (**F**) (*n* = 3; mean ± SD; * *P* < 0.05; ** *P* < 0.01; Student's *t*-test)

To determine how AQP5 affects the expression of Netrin-1 and Slit2, we used the website PROMO (https://alggen.lsi.upc.es/cgi-bin/promo_v3/promo/promoinit.cgi?dirDB=TF_8.3) to make a primary prediction of the TFs of Netrin-1 and Slit2. Their common TFs, and the expression levels of those in RNA sequencing, are shown in the heatmap (Fig. 7A). JunB was significantly increased in the LGs of *Aqp5^−^^/^^−^* mice compared to *Aqp5^+/+^* mice. Furthermore, the results of qPCR confirmed the results of RNA sequencing (Fig. 7B). Western blot analysis showed that JunB was 1.8-fold more highly expressed in *Aqp5^−^^/^^−^* mice LGs compared to *Aqp5^+/+^* mice LGs (Fig. 7C). To identify a binding site that could recruit TFs, the JunB antibody was used in ChIP assays, which showed that JunB can bind to the promoter regions of Netrin-1 and Slit2 (Fig. 7D).

To test the regulatory effect of JunB on Netrin-1 and Slit2 expression levels, we treated primary LG epithelial cells with SR11302, an inhibitor of AP-1. The AP-1 TF is a dimeric complex composed of JUN, FOS, ATF, and MAF protein families, and JunB is a member of JUN protein family. LG epithelial cells treated with SR11302 expressed higher levels of Netrin-1 and lower levels of Slit2 than the DMSO group (Fig. 7E). Western blot analysis showed that in LG epithelial cells, the inhibition of JunB enhanced the Netrin-1 expression level and decreased the Slit2 expression level (Figs. 7F, 7G). The data presented here indicate a suppressive effect of JunB on Netrin-1 and a stimulatory role of Slit2.

## Discussion

DED is mainly characterized by changes in the composition of the tear film. Water, the main component of the tear film, is secreted by the LG. Because LG dysfunction is the primary cause of aqueous tear-deficient DED, significant research efforts have been directed toward determining how we can improve the treatment of DED by focusing on LG, and numerous scientific advancements have been made in this direction. Currently, the transplantation of glands and the use of mesenchymal stem cells (MSCs) for induction or enhancement of in situ LG regeneration seem the most promising strategies.^28^^,^^29^ However, these cannot yet fundamentally relieve the symptoms, meaning many patients with DED still ultimately receive palliative care^30^^–^^32^ due to the condition. Furthermore, the pathological mechanism of the LG in DED is not fully clear. The main contribution of this study is the finding that the activation of JunB mediates the expression of Netrin-1 and Slit2, leading to neural abnormalities in the LGs of *Aqp5^−^^/^^−^* mice.

AQP5 has been demonstrated to be expressed in a variety of glands, including the lacrimal,^33^^,^^34^ salivary,^35^ and submandibular glands,^36^ and plays a key role in gland secretion.^37^^–^^39^ Previous studies have shown the role of AQP5 transport and protein interactions in the dysregulation of salivary gland epithelial cells in Sjögren's syndrome (SS).^40^ The secretion of salivary fluid and protein is mainly dependent on parasympathetic and sympathetic nerves.^41^ As the sampled animals aged, they experienced a decline in both parasympathetic and sympathetic innervation of LG acini.^42^ Our previous study found that AQP5-deficient mice have a stable dry eye phenotype and that AQP5 deficiency leads to pyroptosis in LGs by aggravating the ROS/NLRP3 inflammasome.^27^ However, the effects of AQP5 on glandular nerves are still unclear. In this work, we discovered that the neural distribution of the *Aqp5^−^^/^^−^* mice LGs was reduced overall when compared to those of *Aqp5^+/+^* mice. Sympathetic and sensory neurons were significantly reduced in *Aqp5^−^^/^^−^* mice LGs compared to *Aqp5^+/+^* mice LGs. However, the exact mechanism of the impact of AQP5 on nerves remains unclear.

Neurological abnormalities^43^^,^^44^ occur in DED in addition to ocular surface inflammation and pain. The corneal nerve is most frequently mentioned in DED cases, followed by the retinal nerve, whereas the lacrimal nerve is rarely noted. Unilateral corneal nerve transection induces bilateral ferroptosis of LGs through the VIP/Hif1a/TfR1 pathway, which leads to DED.^45^ Neurotrophins are expressed in the LG and may affect its health and maintenance.^46^ Neurotransmitters and the epidermal growth factor (EGF) family of growth factors regulate the secretion of LGs.^47^ Normal development and functioning depend on the creation of neural networks and connectivity, and axon guidance cues are essential for the formation and development of nerves.^23^^,^^48^^,^^49^ However, little is known about the mechanism of axon guidance cues in the LG. In our study, we found that the LGs of DED model mice had much lower levels of Netrin-1 axon guidance cues and higher levels of those from Slit2. In LGs of *Aqp5^+/+^* mice, Netrin-1 promoted axonal growth of TG cells in *Aqp5^−^^/^^−^* mice. Meanwhile, in the LGs of *Aqp5^−^^/^^−^* mice, Slit2 prevented the TG neurons in *Aqp5^+/+^* mice from growing their axons. However, it is still not clear what causes AQP5 to alter the levels of Netrin-1 and Slit2.

TFs are a group of protein molecules that bind to a specific sequence of a gene to ensure that the target gene is expressed at a specific intensity for a certain time and space. Recent works in drug discovery have revealed that small molecules can directly modulate the function of TFs.^50^^–^^52^ CrebA/Creb3-like TFs are major and direct regulators of the secretory capacity of many glands.^53^ Axonal sprouting is correlated with the expression of Jun, Krox, and Creb TFs in rat and goldfish retinal ganglion cells after optic nerve lesions.^54^ Aire deficiency, meanwhile, alters innervation and leads the secreted axon guidance molecule, SEMA3B, to be expressed by lacrimal ductal cells and significantly increased.^55^ In this work, JunB, as the common TF for Netrin-1 and Slit2, was upregulated in the LGs of *Aqp5^−^^/^^−^* mice and was inhibited by SR11302, an inhibitor of AP-1. After the addition of SR11302, the expression of Netrin-1 increased and that of Slit2 decreased in the LG epithelial cells. These results indicate that JunB activation may mediate the AQP5-induced changes in nerve distribution, and AQP5-induced changes in Netrin-1 and Slit2 may be reversible to a certain extent. Netrin-1 stimulated axonal growth, whereas Slit2 opposed TG axonal growth, and both are regulated by JunB specifically. As to how AQP5 deficiency leads to the upregulation of JunB in LGs, we speculate that the activation of ROS induced by AQP5 deficiency may play a key role. Because the redox-sensitive role of the transcription factor JunB has been uncovered.^56^

A primary nerve culture was used to determine how different factors impact the development of nerve axons more intuitively. The main LG secretion is primarily controlled by sympathetic and parasympathetic nerves, which are reflexively stimulated by sensory nerves on the ocular surface. In prior research, Netrin-1 was found to enhance Dopaminergic (DA) neuron axonal outgrowth, whereas Slit2 inhibited DA neuron extensions.^57^^,^^58^ However, we do not yet know whether Netrin-1 and Slit2 have specific effects on the lacrimal nerve. In the present study, it was discovered that DED dramatically affected the LG sensory and sympathetic nerves. However, due to the technical difficulty and low operability of conducting primary cell extraction on the sympathetic nerves, we chose to do so only for the sensory nerves, which represents a limitation of our research.

**Figure 8.**
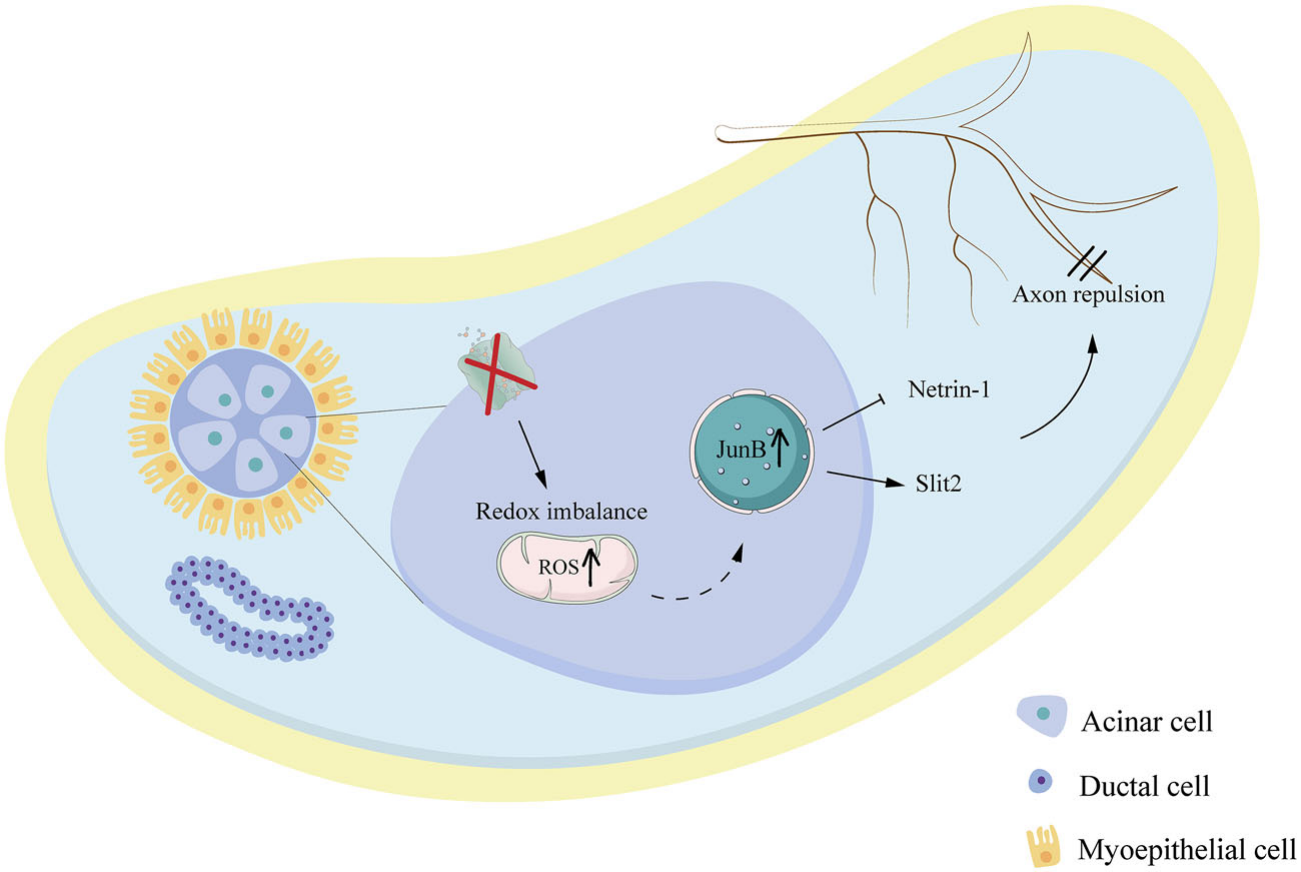
Schema of proposed mechanisms of axonal changes in LGs induced by AQP5 deficiency.

Taken together, our study confirms the decisive role of AQP5 in deciding the neural fate of the LG. We have outlined the specific mechanism by which AQP5 affects the distribution of LG's nerves, that is, by affecting the level of JunB in the LG and then regulating the axon guidance signals of Netrin-1 and Slit2 (Fig. 8). We suggest that AQP5 should be considered a key target for the treatment of DED. The reduction in LG nerve distribution can be alleviated by inhibiting JunB, which provides a new direction for the treatment of DED.

## Supplementary Material

Supplement 1

Supplement 2

Supplement 3

Supplement 4

Supplement 5

Supplement 6

Supplement 7

Supplement 8

Supplement 9

Supplement 10

## Supplementary Material

**Supplementary Video 1**. The 3D assessment of neural distributions in the LG of *Aqp5^+/+^* mouse, related to Figure 1Ai.

**Supplementary Video 2**. The 3D assessment of neural distributions in the LG of *Aqp5^−^^/−^* mouse, related to Figure 1Ai.

**Supplementary Video 3**. The 3D assessment of sympathetic neural distributions in the LG of *Aqp5^+/+^* mouse, related to Figure 1Aii.

**Supplementary Video 4**. The 3D assessment of sympathetic neural distributions in the LG of *Aqp5^−^^/−^* mouse, related to Figure 1Aii.

**Supplementary Video 5**. The 3D assessment of parasympathetic neural distributions in the LG of *Aqp5^+/+^* mouse, related to Figure 1Aiii.

**Supplementary Video 6**. The 3D assessment of parasympathetic neural distributions in the LG of *Aqp5^−^^/−^* mouse, related to Figure 1Aiii.

**Supplementary Video 7**. The 3D assessment of sensory neural distributions in the LG of *Aqp5^+/+^* mouse, related to Figure 1Aiv.

**Supplementary Video 8**. The 3D assessment of sensory neural distributions in the LG of *Aqp5^−^^/−^* mouse, related to Figure 1Aiv.
