# Supplementary material for: Regulation of Axon Guidance by Slit2 and Netrin-1 Signaling in the Lacrimal Gland of Aqp5 Knockout Mice
Source: Invest Ophthalmol Vis Sci. 2023 Sep 14;64(12):27. doi: 10.1167/iovs.64.12.27 (PMC10506685; doi:10.1167/iovs.64.12.27)
Supplement: Supplement 1 [file iovs-64-12-27_s001.pdf]

Supplementary Figure 1: (A) Representative immunofluorescence images of LG tissue labled with DAPI (blue) and AQP5(green). Scale bars: 20μm.

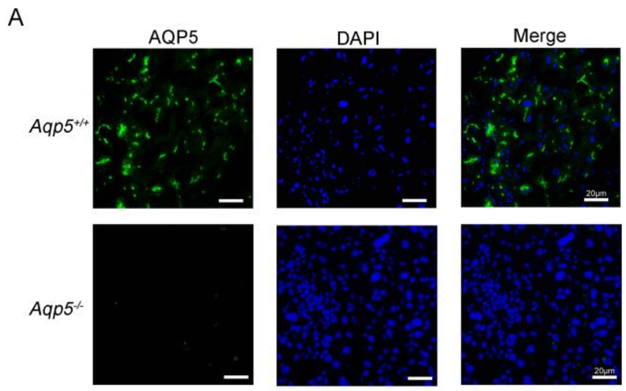

Supplementary Figure 2: (A) Retrograde transport of Fluoro-Glod^{TM} in the LGs ang TGs following a subconjunctival injection. Scale bars: 50μm.

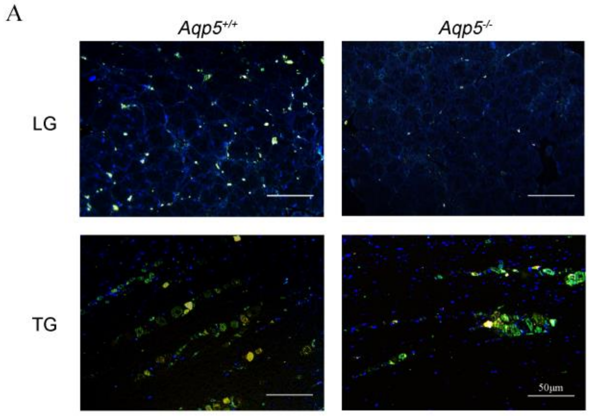

Supplementary Figure 3: The volcano plot for all expressed genes. Differentially expressed genes (FDR < 0.05) between Aqp5$^{+/+}$ and Aqp5$^{-/-}$ LGs were presented with blue (down) and red (up).

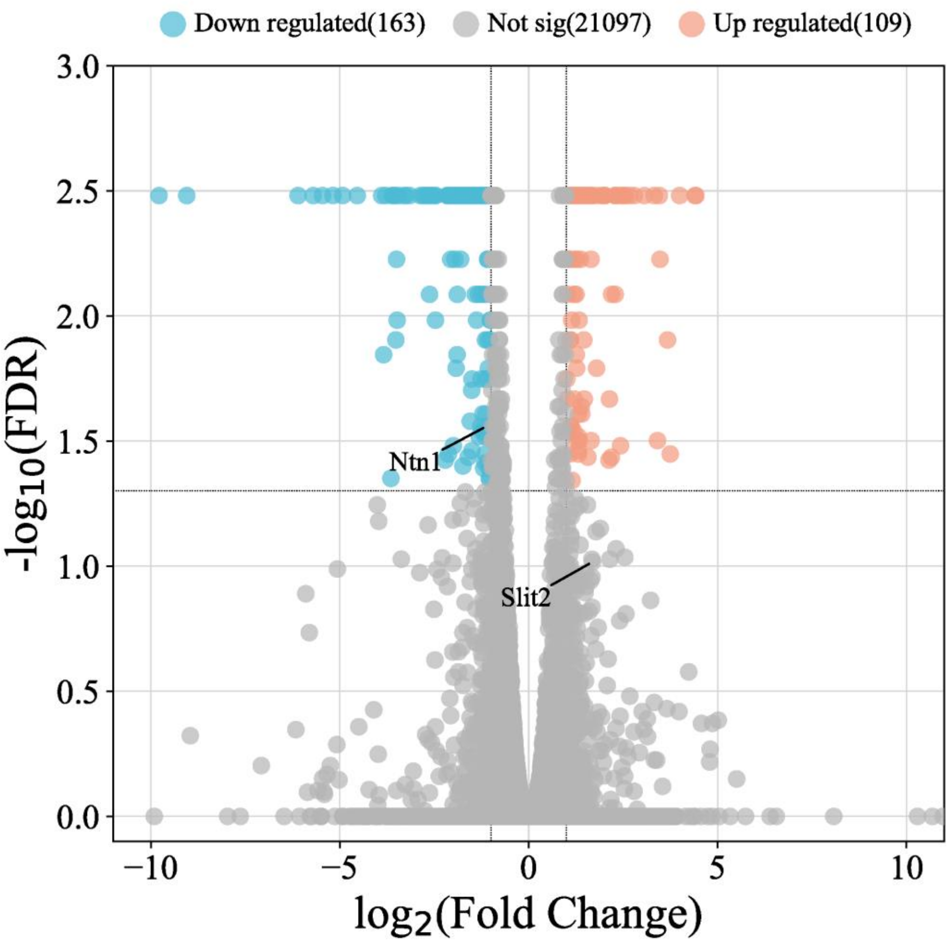

Down regulated(163)
Not sig(21097)
Up regulated(109)
3.0
2.5
2.0
1.5
1.0
0.5
0.0
-log10(FDR)
Ntn1
Slit2
−10
−5
0
5
10
log2(Fold Change)
